# An RCT protocol of varying financial incentive amounts for smoking cessation among pregnant women

**DOI:** 10.1186/1471-2458-12-1032

**Published:** 2012-11-27

**Authors:** Marita Lynagh, Billie Bonevski, Rob Sanson-Fisher, Ian Symonds, Anthony Scott, Alix Hall, Christopher Oldmeadow

**Affiliations:** 1 Priority Research Centre for Health Behaviour, Faculty of Health, The University of Newcastle & Hunter Medical Research Institute, Callaghan, NSW, Australia; 2 School of Medicine and Public Health, The University of Newcastle & Hunter Medical Research Institute, Callaghan, NSW, Australia; 3 Melbourne Institute of Applied Economic Social Research, The University of Melbourne, Parkville, VIC, Australia; 4 The University of Newcastle, West Wing HMRI Building, University Drive, Callaghan, NSW, 2308, Australia

**Keywords:** Smoking cessation, Financial incentive, Pregnancy

## Abstract

**Background:**

Smoking during pregnancy is harmful to the unborn child. Few smoking cessation interventions have been successfully incorporated into standard antenatal care. The main aim of this study is to determine the feasibility of a personal financial incentive scheme for encouraging smoking cessation among pregnant women.

**Design:**

A pilot randomised control trial will be conducted to assess the feasibility and potential effectiveness of two varying financial incentives that increase incrementally in magnitude ($20 vs. $40AUD), compared to no incentive in reducing smoking in pregnant women attending an Australian public hospital antenatal clinic.

**Method:**

Ninety (90) pregnant women who self-report smoking in the last 7 days and whose smoking status is biochemically verified, will be block randomised into one of three groups: a. No incentive control group (n=30), b. $20 incremental incentive group (n=30), and c. $40 incremental incentive group (n=30). Smoking status will be assessed via a self-report computer based survey in nine study sessions with saliva cotinine analysis used as biochemical validation. Women in the two incentive groups will be eligible to receive a cash reward at each of eight measurement points during pregnancy if 7-day smoking cessation is achieved. Cash rewards will increase incrementally for each period of smoking abstinence.

**Discussion:**

Identifying strategies that are effective in reducing the number of women smoking during pregnancy and are easily adopted into standard antenatal practice is of utmost importance. A personal financial incentive scheme is a potential antenatal smoking cessation strategy that warrants further investigation.

**Trial registration:**

Australian New Zealand Clinical Trials Registry (ANZCTR) number: ACTRN12612000399897

## Background

### Smoking during pregnancy is a major public health issue

Smoking rates during pregnancy vary considerably, within and between countries [1-4]. For example, recent data illustrates large variations in smoking rates at the end of pregnancy across England, ranging from 3% to 30% [4]. Similarly, the European perinatal health report have detailed smoking rates during the second trimester from 5% in Lithuania to 22% in France [2]. In 2009, approximately 15% of Australian women smoked during their pregnancy [3]. Rates are known to be higher in certain subpopulations such as lower socioeconomic, indigenous and ethnic minority groups [1,5]. Smoking during pregnancy is directly associated with a number of serious, harmful effects to the baby, including preterm delivery, low birth weight and perinatal mortality [6,7]. Babies born to mothers who smoke during their pregnancy are also more likely to require special care or neonatal intensive care compared to mothers who do not smoke [6].

### Current prenatal smoking cessation interventions are often not adopted into standard care

Despite a recent Cochrane review [8] reporting an absolute difference of 6% reduction in smoking in late pregnancy following cessation interventions, very few are adopted into standard care. Furthermore, pregnant women who do continue to smoke may not receive support to quit [9]. For example, one Australian study found that almost three-quarters of pregnant smokers in one hospital did not receive smoking cessation advice at 30 weeks gestation [9]. Time restraints, competing pressures, staff attitudes, lack of training or skill, administrative barriers and lack of acceptable interventions for women and staff were identified as some of the barriers affecting implementation of smoking cessation into standard antenatal care [5].

### Difficulties in accurately verifying women’ smoking status

Due to social stigma and social desirability, women often underreport their current smoking behaviour, making it difficult for clinic staff to accurately verify women’s smoking status [1]. For example a recent study indicated underestimations of self-reported smoking status during pregnancy by as much as 25% [10]. The incorporation of simple, rapid, biochemical validation methods of patients’ smoking status, may help to overcome this problem. For instance, a study of family practice patients illustrated validity in using a semi-quantitative dipstick assay (NicAlert®) to rapidly asses participant smoking status through saliva cotinine analysis [11]. This study illustrated high sensitivity (99%) and high specificity (96%) of the simple, 20-minute analysis of saliva cotinine [11].

### Contingent reinforcement is an appropriate strategy for health behaviour change

The use of a contingent reinforcement schedule, such as a Personal Financial Incentive (PFI), whereby individuals are provided a monetary reward for their engagement in a particular behaviour, may be an effective strategy to assist in reducing smoking behaviour. Contingent reinforcement has strong theoretical grounding within behavioural psychology and more specifically the theory of operant conditioning [12]. The lack of relevant health promotion theories being applied to current smoking cessation interventions for pregnant women has been identified as a general criticism of interventions conducted in this area [5]. Contingency management is based on the notion that because behaviour is reinforced by its consequences it can be modified by changing the consequences [12]. There are several guiding principles of contingency management suggested as potentially relevant in treating substance use [13,14] and additional principles which have been identified as effective to PFI by previous literature reviews [15,16]. These include the use of: behavioural contracting; positive reinforcements; frequent and immediate feedback and rewards; and incentives of sufficient and incremental magnitude [14]. The use of financial incentives also has a strong theoretical and empirical grounding in economics, where the monetary transfer influences the ratio of benefits to costs, that in turn influences choice and decision making.

Contingency management approaches have been successfully applied to other health behaviours and several have been effectively translated into real world practice [17]. For example, rewards or reduced premiums have long been utilised by health insurers in the United Kingdom and South Africa to encourage individuals to engage in health promoting behaviours, such as exercise or screening programs [16]. In Australia the Government has successfully incorporated incentive-based programs to influence several health behaviours including, immunisation and population growth [18-20]. These programs resulted in an increase in child immunisation rates from 56% in 1996 to 90% in 2003 [20]; and a significant rise in fertility rates [18,19]. Additionally, the results from one state-based study found no significant differences in fertility rates of socially disadvantaged groups compared to non-disadvantaged groups [19]. The success of such financially based incentives introduced by the government to influence health behaviours provides a strong platform for how a similar program to reduce smoking in pregnant women could be introduced into practice, if found feasible and effective.

### Contingent reinforcement has been found to be effective in facilitating smoking cessation

A recent Cochrane review assessing smoking cessation interventions in pregnant women found that interventions that included an incentive component resulted in a significantly larger effect in point-prevalence abstinence during late-pregnancy, compared to other intervention strategies [15]. Though PFI seems to be a potentially effective method in promoting smoking cessation in pregnant women, very few studies have assessed their effectiveness among pregnant smokers in an Australian antenatal care setting. Additionally, there have been no Australian studies which have assessed the effect of reward size on smoking cessation behaviour. One recent descriptive study found pregnant women had mixed and often unfavourable views regarding the acceptability and potential effectiveness of using PFIs to assist women to quit smoking [21]. However, a significantly higher percentage of smokers reported favourable views on the use of PFI for smoking cessation, highlighting the ambiguity surrounding the acceptability and potential benefit of such a strategy [21]. Consequently, it is important that research is conducted which aims to assess whether a PFI smoking cessation program is feasible in an Australian antenatal care setting.

### Aims

The primary aim of this study is to test the feasibility of a personal financial incentive (PFI)-based intervention at encouraging smoking cessation in pregnant women attending an Australian public hospital for antenatal care. Specifically, the study will assess: (i) consent rates of women to participate in the trial; (ii) loss to follow-up rates of study participants; (iii) compliance with saliva cotinine and hair cotinine analyses for biochemical validation (iv) acceptability of using a touchscreen computer for self-reported smoking status and demographics; (v) acceptability of saliva cotinine and hair cotinine analyses for biochemical validation of smoking status; (vi) acceptability of incentives of varying magnitude ($20 vs. $40) to staff and study participants; and (vii) the perceived barriers and facilitators to using financial incentives in a public antenatal clinic among clinic staff. Additionally, secondary aims include: (i) to assess the potential effectiveness of the varying PFI amounts ($20 vs. $40) in reducing smoking among study participants; (ii) to assess the accuracy of self-report smoking status with saliva and hair cotinine analysis; and (iii) to undertake a cost-effectiveness analysis comparing the changes in costs of the intervention and changes in quit rates between each arm of the trial.

## Methods/design

### Study design

This is a proof-of-concept trial that will employ a randomised control trial design to assess the potential efficacy of personal financial incentives on pregnant women smoking cessation rates; combined with a qualitative analysis to investigate the acceptability of the intervention to participants and clinic staff. The study will involve a baseline survey; eight intervention sessions; and a post-intervention telephone interview of participating women and semi-structured interviews with clinic staff. Self-reported smoking status will be validated through saliva cotinine analysis using NicAlert® semi-quantitative dipstick assay. Continuous or long-term smoking cessation will be additionally assessed through hair analysis of patient’s cotinine levels using gas chromatography – mass spectrometry.

### Setting

The study will take place at the antenatal clinic within a large public teaching hospital in New South Wales, Australia. The antenatal clinic operates a 5 days-a-week service with most women presenting for an average of 6 to 8 clinic visits over the course of their pregnancy. The hospital manages approximately 4,300 deliveries each year with the majority of these women receiving antenatal care predominantly through the hospital antenatal clinic.

### Participants

A sample of ninety (90) consenting pregnant women will be recruited to participate. Women will be considered eligible if they are: (1) aged at least 16 years; (2) presenting for their first antenatal visit; (3) less than 31 weeks gestation; (4) sufficient in English language to complete the survey; and (5) a current smoker (defined as having smoked in last 7 days and a return a positive saliva sample for cotinine). Pregnant women will be ineligible to participate if they have elected to receive shared antenatal care with their GP and/or are considered by clinic staff to have a severe cognitive or psychiatric disorder; currently being treated for chemical dependency other than alcohol or tobacco; or have quit smoking before their first antenatal appointment.

### Randomization

Consenting women will be randomly allocated using block randomisation into one of three groups: control vs. smaller incentive ($AUS20) vs. larger incentive ($AUS40). The unit of randomisation will be the day and session time (i.e. morning or afternoon) of the patient’s first antenatal visit to minimise potential contamination and deception. Randomisation will be performed offsite, by an independent statistician prior to study initiation. Allocation of days of the week to groups will be done using Proc Plan in SAS.

### Intervention

Two intervention arms will be assessed: (1) a $AUD20 incremental personal financial incentive; and (2) a $AUD40 incremental personal financial incentive. Women from both intervention groups will have an opportunity to receive a PFI at eight study intervention sessions contingent upon smoking abstinence. The amount of the monetary reward will begin at the base amount (either $AUD20 of $AUD40) and will increase by the base amount (either $AUD20 of $AUD40) at each follow-up that participants are found to abstain from smoking. Smoking abstinence will be assessed through self-report and confirmed by saliva cotinine analysis. Participants in the $AUD20 intervention group, who quit smoking and maintain cessation for the entire eight intervention sessions, will be potentially eligible to receive a total of $AUD720. Participants in the $AUD40 group will be potentially eligible to receive a total of $AUD1440 if they maintain smoking abstinence for the 8 intervention sessions. If a participant fails to abstain at one intervention session or does not present for a scheduled visit they will not receive the incentive for that period. However, the reward amount these women will be eligible for during their next intervention session will be the amount they missed during their previous session. Their potential reward amount will remain at this amount until they have successfully stopped smoking.

### Procedures

All women who visit the hospital antennal clinic will undergo their usual antenatal interview by the clinic nurse. During this interview patients are asked about their smoking status and basic demographic characteristics. At this time potentially eligible patients will be identified and informed that the study is being conducted within the clinic. If women are interested they will be directed to a Research Assistant (RA) who will be stationed within the clinic. The RA will provide interested women with information describing the study. If patients would like to take part in the study the RA will obtain their written informed consent. Women will then be asked to provide a saliva sample to confirm their smoking status and complete a 15-minute baseline Touchscreen computer survey, which will also ask for their on-screen consent.

If a woman’s initial antenatal appointment has been randomised to one of the two intervention groups, the RA will verbally explain the details of their incentive scheme, including, the schedule for monitoring, contingencies being offered and the rules of payment. All participants will be provided with a study package to take home with them, which will contain information about the study, referrals to Quit smoking self-help documents and a Quit telephone help number (provided by clinic midwives) and a letter of support to pass onto their partner. Consenting women will be asked to attend 8 intervention sessions, at which participants in the two intervention groups will be eligible to receive a reward. The 8 intervention sessions will occur approximately fortnightly, during participants’ scheduled antenatal appointments.

During each of the 8 intervention sessions, all women will be asked to indicate their current smoking status and whether they have quit smoking in the last 7 days. Women will also be asked to provide a saliva sample which will be analysed to confirm their self-report smoking abstinence. Those women in the intervention groups who self-report non-smoking and return a negative cotinine reading (level 0) will be provided with their monetary incentive. A similar follow-up procedure will occur seven more times (i.e. a total of 8 intervention sessions) during women’s scheduled antenatal appointment.

During their eighth or final intervention session (i.e. the last follow-up where women are eligible for a reward) women will be asked to provide a hair sample to allow for cotinine analysis using gas chromatography – mass spectrometry. The hair analysis will allow for long-term assessment of women’s smoking status. Women will also be asked to consent to a post-intervention study approximately six weeks after delivery. Women who consent will be contacted via telephone. A short semi-structured telephone interview will be conducted assessing women’s acceptability of the intervention and their current smoking status. A similar interview will be conducted with clinic staff to assess their acceptability of, and feedback on, the program. Both participants and staff will be asked to provide feedback on whether they believe the intervention was useful in increasing smoking abstinence in pregnant women, whether the monetary reward was sufficient and/or delivered in an appropriate manner, whether they believe this intervention could be easily introduced into standard care and whether they experienced any problems with the intervention.

### Primary outcome measures

The RA will maintain detailed records documenting: (1) the number of eligible women asked to take part in the research study and how many of these women consent to take part; (2) the number of participants who withdraw from the study before study completion; and (3) the number of women who complete or refuse to provide a saliva and/or hair sample for cotinine analysis. This data will be used to measure the following three primary outcomes: (i) consent rates; (ii) loss to follow-up rates of study participants and (iii) participant compliance with saliva and hair cotinine analyses for biochemical validation of smoking status

Semi-structured interviews will be conducted with both participants and clinic staff. Women will be asked a serious of previously published and study specific questions about their experiences and perceptions of the intervention trial. Specifically, six questions previously published by Bryant et al. [22] will be included in the interviews with study participants, to assess their acceptability of using a touchscreen computer for self-reported smoking status and demographics. Five (5) items previously adapted by the researchers to assess pregnant women’s acceptability of PFI as a strategy to decrease smoking in pregnant women [21], will be included in both participant and staff interviews. These questions will be used to investigate the acceptability of incentives of varying magnitude to staff and study participants. Open-ended questions will also be included in both participant and staff interviews to assess their thoughts on the acceptability of using saliva and hair cotinine analysis for biochemical validation of smoking status, as well as any perceived barriers and facilitators to using financial invectives.

### Secondary outcome measures

To measure participants’ self-reported smoking status women will complete one baseline and eight follow-up self-report touchscreen computer surveys. The computer survey was programed using Digivey survey software [23]. Use of computer surveys to assess smoking status has been shown to be a reliable and accurate method of data collection [24], as well as being reported as enjoyable and easy to complete by most participants [22].Questions assessing participants’ demographic characteristics and factors previously found to be associated with smoking status will also be included. The following questions will be included in the self-report, touchscreen computer surveys:

*Baseline survey*:

• *Smoking status and history* will be assessed through standardised questions or questions adapted from previous research [22,25-32] with many items based on the previous the work of Bryant et al. [22,26,27] Items will include: current smoking status [22,26], number of cigarettes currently smoked per day [30], smoking history, previous quit attempts, reductions in smoking [25,26], strategies used to assist in previous quit attempts [26,27], readiness/stages of change [29,31], financial stress [33,34], whether they smoked during any previous pregnancies [32]and whether they attempted to quit or reduced smoking during any previous pregnancies [26,32]. Questions assessing women’s exposure to environmental smoke will also be included [25,26,32].

• *Demographic characteristics*: women will be asked to answer questions relating to their: age, date of birth, highest level of education, Aboriginal or Torres Strait Islander status, current household income, marital status, number of previous pregnancies, gestation period, postcode, number of adults and children (<18 years) residing in their household.

• *The Patient Health Questionnaire2* (*PHQ2*) [35]: Will be included as a short, simple measure of participants’ levels of depression. The PHQ2 consists of two questions, which assess respondent’s level of depressed mood in the previous 2 weeks [35]. Respondents rate the frequency of depressed mood and anhedonia on a four point likert scale, ranging from 0 (“not at all”) to 3 (“nearly every day”) [35]. Total score on the PHQ2 range from 0 to 6. A score of 4 has recommended as an appropriate cut-point for screening for possible depressed mood in pregnant women [36]. The PHQ-2 has evidence of criterion validity; as well as acceptable specificity (92%) and sensitivity (83%) [35]. Previous research has found a relationship between depression and smoking status [37,38].

• *Economic questions*: Women will be asked to a number of questions relating to their personal out-of-pocket costs, time costs and health care utilisation incurred as a result of the intervention.

• *Social characteristics*: women will be asked whether their partner or others within their household smoke.

*Follow*-*up surveys*: During each of the follow-up visits, women will be asked to complete a brief touchscreen computer survey using a number of items from the baseline survey. Questions will assess their current smoking status, number of cigarettes smoked per day (if they have not abstained), changes in smoking behaviour, partners’ current smoking status, exposure to environmental smoke, the number of quit attempts made, gestation age; the PHQ2 and efforts to engage with quit smoking support services.

The following two biochemical analyses will be used to validate women’s self-reported smoking status:

1. *Saliva cotinine analysis*: will be used to biochemically validate self-reported smoking status for all participants at baseline and each of the eight intervention sessions using the NicAlert® semiquantitative dipstick assay. A previous study analysing 167 family practice patients using NicAlert® illustrated acceptable levels of sensitivity (99%) and specificity (96%) [11].Cotinine analysis with the NicAlert® dipstick takes approximately 20 minutes and can be performed by untrained persons, highlighting the potential utility of such a test in clinical practice .

2. *Hair cotinine analysis*: will be undertaken during the eighth or final intervention session. Participants will be asked to provide a small sample of hair taken from the nape of the neck. Samples will be transported to an off-site laboratory for analysis. Hair cotinine analysis can reliably quantify long-term exposure to tobacco, with each centimetre of scalp hair representing approximately 1 month of past exposure [39]. Gas chromatography – mass spectrometry will be used to analyse hair cotinine levels, as it is considered the standard reference in analysis of cotinine [40]. A cut-point of 0.2 ng/mg has been found to distinguish between pregnant active smokers and passive or unexposed with high levels of sensitivity (91%), specificity (94%) and test accuracy (91.7%) [40]. We will also validate hair cotinine analysis as a measure of smoking status by comparing its accuracy against women’s self-report smoking status and saliva cotinine throughout the study.

#### Economic outcomes measures

The feasibility of undertaking a cost-effectiveness analysis will also be undertaken. This will include the collection of data on the costs of the interventions that would include the amount of money received by each participant, and the costs of administering the payments in terms of staff time and other resources used. The intervention may also encourage patients to attend their antenatal visits, and potentially other health care visits associated with their pregnancy, more often than the control group, and so data will also be collected on patients’ utilisation of health services throughout the study. The feasibility of obtaining these data from Medicare, PBS, and hospital separations data, including obtaining patients’ consent, will be investigated and compared to gathering this data directly from patients using a standard questionnaire administered at each visit. The costs incurred by patients are also likely to be affected by the intervention. This will include savings in purchasing cigarettes, and differences in out of pocket, travel and time costs of attending if the intervention influences health care utilisation. These data will be collected from a short patient survey administered at each visit. Out of pocket costs for GP visits and prescribed medication can also be collected from MBS and PBS records. The effectiveness data on quitting will be collected as mentioned above, and combined with the data on costs. Incremental cost-effectiveness ratios (ICERs) will be calculated [41].

### Sample size

A convenience sample size totalling 90 women will be recruited, with 30 women randomised to each of the three study groups. As this is a proof-of-concept trial, the sample size will not be adequately powered to detected small differences however a sample size of 30 women in each group will allow for detection of a difference in smoking rates between groups of 24%, with 5% significance level and 68% power. Based on previous studies, this will be sufficient to provide an estimate of the difference in proportions between groups for the following primary outcomes: (i) consent rates; (ii) lost to follow-up rates; (iii) participant compliance to saliva and hair cotinine analysis; (iv) acceptability of incentives and validation measures, and the secondary outcome variable, smoking cessation. To allow for a 20% drop-out rate, a consent rate of 60% and 10% of women being ineligible, 208 pregnant women who smoke will be approached. Based on a previous study conducted by the investigators, approximately 17% of women seen at this clinic are self-reported smokers [21].

### Analysis

Baseline demographic and social characteristics of patients in all the three treatment groups will be summarised separately. Continuous data will be expressed as mean values with standard deviations (SD), and categorical data will be presented as counts with percentages.

### Primary outcomes

Proportions and frequencies will be calculated to assess the following outcomes: (i) participant consent rates; (ii) loss to follow-up rates; and (iii) participant compliance with saliva and hair cotinine analysis. Proportions and frequencies will also be calculated for all closed-ended questions included in the study participant and clinic staff interviews to allow investigation of: (iv) participant acceptability of touchscreen computers for self-reported smoking rates and demographics; and (v) acceptability of incentives to staff and study participants. Differences between experimental groups in the proportion of each of these five primary outcomes and the secondary outcome (proportion quit smoking) will be assessed through Fisher’s exact test. Differences between participant subgroups and these outcomes will also be assessed using Fisher’s exact test. Although p-values from the above will be reported, the focus will be on providing 95% confidence intervals around point estimates for comparisons of all three treatment arms.

Qualitative analysis will be performed on open-ended questions included in participant and staff interviews investigating the following primary outcomes: (vi) acceptability of saliva and hair cotinine analyses for biochemical validation of smoking status; and (vii) perceived barriers and facilitators to using financial incentives. All interviews will be transcribed verbatim and a thematic analysis will be performed independently by two of the research team members.

### Secondary outcomes

#### Quit rates

Although not adequately powered for detecting small differences in the secondary outcomes, Fisher’s exact test will be used to detect potential differences in smoking cessation rates between groups and to demonstrate potential effectiveness of the intervention. Differences between experimental groups in the secondary outcome - PHQ2 will be assessed using the Kruskal-Wallis test. Fisher’s exact tests will be used to compare conditions on post-partum smoking rates at 6 weeks after delivery.

#### Validation analysis of saliva and hair cotinine compared to self-report smoking rates

Agreement between self-reported smoking status and the cotinine saliva test strip will be assessed using the Kappa statistic. Agreement between self reported smoking status and hair cotinine results will also be assessed using the Kappa statistic.

#### Economic analysis

Quantities of resources used (e.g. number of visits) will be combined with their unit costs and calculated from existing sources using standardised methods. Differences in the average cost per patient in each arm of the trial will be compared with differences in quitting rates. These are used to calculate incremental cost-effectiveness ratios (ICERs) for each intervention arm compared to the control arm.

### Ethics and safety approval

The study will be conducted in accordance with the Helsinki Declaration and has received ethical approval from both the Hunter New England Human Research Ethics Committee (Ref No: 11/09/21/4.05) and the University of Newcastle Human Research Ethics Committee (Ref No: H-2012-0047). Safety approval was also obtained from the University of Newcastle Health and Safety Review Committee (Ref: 26/2012).

## Discussion

The harmful effects of smoking during pregnancy are well known. Identifying strategies that are effective in reducing the number of women smoking during pregnancy and that are easily adopted into standard antenatal practice is of utmost importance. Despite numerous intervention studies attempting to address this issue very few have been successfully adopted into standard care. Given the past success of Personal-Financial Incentives in changing people’s health behaviours and being incorporating into routine care on a large scale, a similar strategy to assist women in quitting smoking seems to be a reasonable avenue for investigation. If the use of PFI as a strategy to reduce smoking during pregnancy is viable and acceptable to women and clinic staff, as well as illustrating promise of effectiveness, such a strategy has the potential to fill the current void in effective smoking cessation programs for pregnant women.

## Abbreviations

PFI: Personal Financial Incentive; RA: Research Assistant; RCT: Randomised control trial.

## Competing interests

The authors declare that they have no competing interests.

## Authors’ contributions

ML, RSF and BB developed the original protocol and intervention concept. IS provided clinical advice towards the development of the intervention and protocol, and assisted in establishing collaborations with the hospital clinic. AS developed, refined and wrote the economic component of the study. ML, BB and AH further refined the study protocol and intervention components and methods. CO provided statistical input and finalised the writing of the statistical methods. ML and AH were responsible for drafting the manuscript. All authors reviewed and approved the final manuscript.

## Authors’ information

ML is a senior lecturer and researcher in the area of health behaviour, working on research projects relating to cancer survivorship, teaching and health behaviour interventions. RSF is a Laureate Professor of health behaviour. He successfully combines behavioural and public health approaches to health promotion, health service evaluation and cancer control. BB has experience in health behavioural research, particularly in the areas of cancer prevention and cancer control. IS is a Senior Staff Specialist in Obstetrics & Gynaecology; with previous experience in the design and funding of research protocols. AS is a health economist with a PhD in health economics. He has worked in economics in Australia and internationally. AH is a PhD student with previous experience in the area of cancer survivorship and some experience in prevention. CO is a biostatistician and postdoctoral research fellow with the Hunter Medical Research Institute with previous experience in statistical analysis of public health research projects.

## Pre-publication history

The pre-publication history for this paper can be accessed here:

http://www.biomedcentral.com/1471-2458/12/1032/prepub
